# Gut Microbiota Dysbiosis and Risk of Venous Thromboembolism: A Systematic Review

**DOI:** 10.3390/microorganisms14051059

**Published:** 2026-05-08

**Authors:** Anabel Franco-Moreno, Cristina Lucía de Ancos-Aracil, Ana Martínez-Casa-Muñoz, Juan Torres-Macho, Eva Ruiz-Navío, Ana Bustamante-Fermosel, María Hornero-Vázquez, Miguel Ángel Casado-Suela

**Affiliations:** 1 Venous Thromboembolism Unit, Hospital Universitario Infanta Leonor, Gran Via del Este Avenue, 80, 28031 Madrid, Spain; 2 Department of Internal Medicine, Hospital Universitario Infanta Leonor, Gran Via del Este Avenue, 80, 28031 Madrid, Spain; 3 Department of Medicine, Universidad Complutense de Madrid, 28040 Madrid, Spain; 4 Venous Thromboembolism Unit, Department of Internal Medicine, Hospital Universitario de Fuenlabrada, 28942 Madrid, Spain; 5 Hospital Universitario Gregorio Marañón, 28007 Madrid, Spain; 6 Hospital Universitario Infanta Leonor, 28031 Madrid, Spain

**Keywords:** deep vein thrombosis, dysbiosis, gut microbiota, hypercoagulable status, immune activation, microbiome, pulmonary embolism, systematic review

## Abstract

Gut microbiota dysbiosis has been proposed as a potential contributor to venous thromboembolism (VTE), although its clinical relevance remains uncertain. This review was conducted following the PRISMA 2020 guidelines. We conducted a systematic review of studies evaluating the association between gut microbiota dysbiosis and VTE. MEDLINE/PubMed, Web of Science, EMBASE, and Scopus were searched from the inception to February 2026. Observational studies and Mendelian randomization (MR) analyses assessing microbiota composition, dysbiosis, or related metabolites in relation to VTE were included. Risk of bias was assessed using established tools. Thirteen studies were included (five observational and eight MR). Observational evidence showed heterogeneous findings. Microbial metabolites such as trimethylamine N-oxide and lipopolysaccharides were associated with prothrombotic profiles in some studies, but no consistent association with VTE risk or recurrence was observed. The differences in microbiota composition were reported, although based on small populations. MR analyses identified microbial taxa with potential protective or risk associations, but most findings were modest and not robust after multiple testing correction. Gut microbiota dysbiosis may contribute to VTE through inflammatory and metabolic pathways; however, current clinical evidence is limited and inconsistent. Further prospective studies are needed to clarify causality and clinical implications.

## 1. Introduction

The human gastrointestinal tract harbors trillions of microbial cells, an essential component of our healthy physiological ecosystem [1]. The healthy adult gut microbiota is predominantly composed of two major phyla, Bacteroidetes and Firmicutes, with smaller proportions of Actinobacteria, Proteobacteria, and Verrucomicrobia [2]. In addition, it includes methanogenic archaea (primarily *Methanobrevibacter smithii*), eukaryotes (predominantly yeasts), and a diverse population of bacteriophages. The adult gut microbiota is relatively stable; however, the specific species and strains vary markedly between individuals. Indeed, each person’s microbial composition is unique [2].

**Figure 1:**
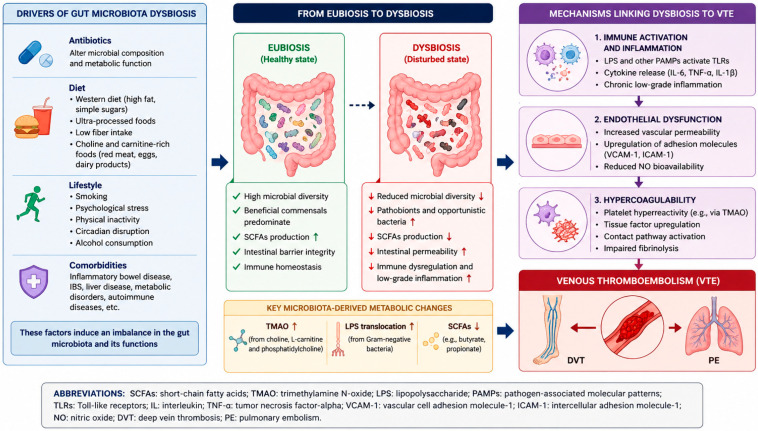
Factors and mechanisms linking gut microbiota dysbiosis to venous thromboembolism. The arrows illustrate the directional relationship between the different stages and mechanisms represented in the figure. Blue horizontal arrows indicate progression from gut microbiota eubiosis to dysbiosis and its subsequent contribution to venous thromboembolism development. Vertical arrows within the right panel represent the sequential interplay between inflammation, endothelial dysfunction, and hypercoagulability. Upward arrows (↑) indicate an increase or upregulation, whereas downward arrows (↓) indicate a decrease or impairment of the corresponding process or metabolite.

Intestinal dysbiosis refers to an imbalance in the gut microbial ecosystem, characterized by alterations in microbial diversity, changes in the relative abundance of specific microbial populations, disruption of microbial-derived metabolites, and impairment of intestinal barrier function. It results from a multifactorial interplay of exogenous and endogenous determinants (Figure 1). Antibiotics are a well-established driver, inducing significant alterations in microbial composition and metabolic function [3,4]. Diet plays a central modulatory role. Western dietary patterns—characterized by high intake of fats and simple sugars—as well as consumption of ultra-processed foods, are associated with reduced microbial diversity and functional instability, while low fiber intake exacerbates microbiome disruption and delays recovery after perturbations [4,5]. Additionally, foods rich in choline and carnitine, such as red meat, eggs, and dairy products, may promote the generation of prothrombotic metabolites. In parallel, lifestyle-related factors—including psychological stress, smoking, physical inactivity, circadian disruption, and alcohol consumption—contribute to microbial imbalance [6]. Dysbiosis is also associated with multiple chronic conditions, including inflammatory bowel disease, irritable bowel syndrome, liver disease, metabolic disorders, and autoimmune diseases, with evidence suggesting a bidirectional relationship [7].

Emerging evidence suggests that gut microbiota dysbiosis may play an underappreciated role in the pathogenesis of venous thromboembolism (VTE) through multiple interconnected mechanisms involving microbial composition, intestinal barrier function, bacterial metabolites, immune modulation, and activation of coagulation pathways [8,9,10]. Dysbiosis is characterized by a reduction in beneficial commensal bacteria and an overrepresentation of potentially pathogenic taxa, leading to a decreased production of short-chain fatty acids (SCFAs) and impaired intestinal barrier integrity. Among microbial metabolites, trimethylamine N-oxide (TMAO) has emerged as a key prothrombotic mediator. Derived from gut microbial metabolism of dietary choline, phosphatidylcholine, and L-carnitine, TMAO enhances platelet reactivity by modulating intracellular calcium signaling, it promotes thrombus formation, and it has been independently associated with thrombotic events [11]. In addition, TMAO induces endothelial expression of tissue factor and adhesion molecules, such as VCAM-1, and activates inflammatory pathways, including the NLRP3 inflammasome. Lipopolysaccharides (LPS), components of Gram-negative bacteria, represent another critical link; disruption of intestinal barrier integrity facilitates their translocation into the circulation, leading to low-grade endotoxemia. LPS activates Toll-like receptors on endothelial cells and platelets, triggering inflammation, activating the coagulation cascade, and contributing to a prothrombotic phenotype [8]. Dysbiosis also influences immune regulation, including alterations in immune cell-mediated pathways and reduced production of short-chain fatty acids, which are key immunomodulatory metabolites [12]. In addition, gut microbiota plays a relevant role in bile acid metabolism, which may exert context-dependent effects on coagulation and platelet function. These processes converge to promote endothelial dysfunction, platelet activation, and increased tissue factor expression, ultimately resulting in a hypercoagulable state. Collectively, these interconnected pathways provide a mechanistic link between alterations in the gut microbiota and VTE risk, highlighting the microbiome as a potential therapeutic target (Table 1).

Given the current lack of knowledge in this field, this systematic review aims to evaluate the available evidence on the association between gut microbiota composition, microbiota-derived metabolites, and venous thromboembolism, as well as their potential mechanistic links.

## 2. Materials and Methods

This systematic review was conducted and reported in accordance with the Preferred Reporting Items for Systematic Reviews and Meta-Analyses (PRISMA) 2020 guidelines.

### 2.1. Literature Search

A comprehensive search was conducted in MEDLINE/PubMed, Web of Science, EMBASE, and Scopus to identify studies evaluating the association between gut microbiota dysbiosis and VTE. The search covered the period from database inception to February 2026. The search strategy combined Medical Subject Headings (MeSH) and free-text terms related to gut microbiota, VTE, and microbiota-derived metabolites and inflammatory pathways, including “gut microbiota”, microbiota, microbiome, dysbiosis, “venous thromboembolism”, VTE, “deep vein thrombosis”, DVT, “pulmonary embolism”, PE, “trimethylamine N-oxide”, TMAO, lipopolysaccharide, LPS, inflammation, and “immune activation”, using Boolean operators (AND/OR), and it was adapted to each database. The search was restricted to studies involving human subjects, with no language limitations. In addition, the reference lists of included articles and relevant reviews were manually screened to identify further eligible studies.

### 2.2. Inclusion and Exclusion Criteria

The inclusion criteria were as follows: (a) studies involving adult human populations; (b) original studies evaluating the association between gut microbiota dysbiosis and VTE, including observational designs (cohort, case–control, or cross-sectional) and Mendelian randomization (MR) analyses; (c) studies assessing microbiota composition, dysbiosis, or related metabolites (e.g., trimethylamine N-oxide); and (d) studies reporting VTE outcomes, including deep vein thrombosis (DVT) or pulmonary embolism (PE), objectively confirmed by standard diagnostic methods. The exclusion criteria were as follows: (a) review articles, editorials, and letters without original data; (b) duplicate publications; (c) studies not involving human subjects; and (d) studies without sufficient data to assess the association of interest. Conference abstracts and case series were eligible for inclusion if they provided relevant data.

### 2.3. Article Selection

Two reviewers (M.A.C.S and A.F.M.) independently screened titles and abstracts of all retrieved records, followed by full-text articles. A third reviewer (J.T.M.) resolved discrepancies. The selection process was aided by a standardized screening form.

### 2.4. Data Extraction and Analysis

Using a standardized form, the data from the studies included were extracted by A.F.M. and M.A.C.S., and reviewed by C.L.A.A. The following information was collected: (a) study setting (country, year of publication, and data collection period); (b) study population characteristics (sample size, age, sex, and ancestry when available); (c) Mendelian randomization design (one-sample, two-sample, bidirectional, or multivariable MR) and data sources (e.g., MiBioGen consortium, UK Biobank, and FinnGen); (d) exposure definition (gut microbiota taxa) and outcomes (VTE); and (e) main results, including effect estimates (odds ratios and corresponding confidence intervals when available), *p*-values, heterogeneity (Cochran’s Q), and pleiotropy (MR-Egger intercept). Effect measures were extracted as reported in the original studies, including odds ratios (ORs) with corresponding 95% confidence intervals (CIs), hazard ratios (HRs), and additional measures such as area under the curve (AUC), correlation coefficients, and differences in metabolite levels, depending on study design. A qualitative synthesis of the included studies was performed. Due to substantial heterogeneity across studies in terms of design, populations, and reported outcomes, a quantitative synthesis (meta-analysis) was not considered appropriate. Sensitivity analyses were not conducted due to the lack of quantitative synthesis. Given the absence of a meta-analysis, formal investigation of heterogeneity was not performed. Data pooling was performed using R software, version 4.3.2 (R Foundation for Statistical Computing, Vienna, Austria), when appropriate.

### 2.5. Assessment of Risk of Bias in the Studies

Risk of bias was assessed according to study design. Observational studies were evaluated using the Newcastle–Ottawa Scale. Mendelian randomization studies were assessed using a structured approach adapted for instrumental variable analyses, considering the core assumptions of relevance, independence, and exclusion restriction. Specifically, we evaluated (a) the strength of the genetic instruments when reported (e.g., F-statistics), (b) potential confounding and population stratification, and (c) the presence of horizontal pleiotropy (e.g., MR-Egger intercept, MR-PRESSO). In addition, heterogeneity across instruments (Cochran’s Q) and the consistency of results across sensitivity analyses (inverse variance weighted, MR-Egger, and weighted median) were assessed. The overall risk of bias was qualitatively categorized as low, moderate, or high based on these domains. Judgments were reached by consensus among three reviewers (A.F.M., M.A.C.S., and C.L.A.A).

The certainty of the evidence was assessed qualitatively using a GRADE-based approach, considering study design, consistency, directness, precision, and risk of bias.

### 2.6. Outcomes

The outcomes of interest included gut microbiota composition, microbiota-derived metabolites, and their association with VTE risk.

## 3. Results

### 3.1. Study Selection

**Figure 2:**
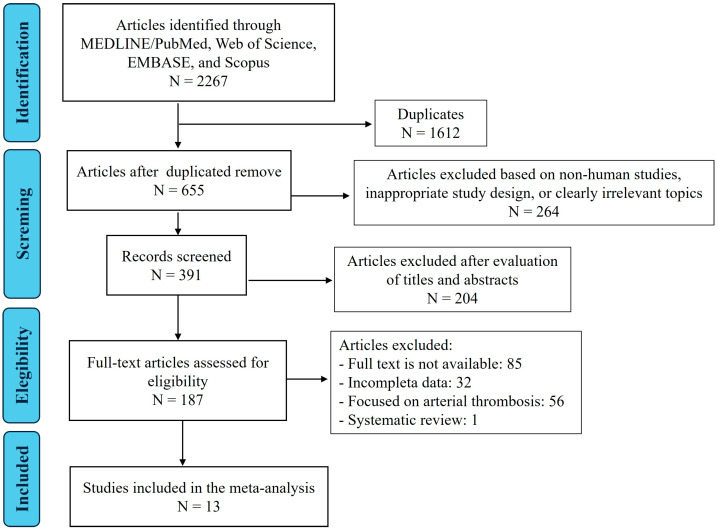
Flow chart of the study selection for the review.

A total of 2267 studies were initially identified, and 655 remained after duplicates were removed. Of these, 264 records were excluded after preliminary screening, based on study type, non-human studies, or clearly irrelevant topics, and 391 records were subsequently screened by title and abstract. A total of 187 full-text articles were assessed for eligibility. Finally, 13 studies were included in the systematic review [13,14,15,16,17,18,19,20,21,22,23,24,25] (Figure 2).

### 3.2. Characteristics of the Included Studies’

The main characteristics, exposures, methodological features, and outcomes of the included studies are presented in Table 2.

#### 3.2.1. Observational Studies

Five observational studies evaluated the association between gut microbiota, microbial metabolites, and VTE. A prospective cohort study included 859 patients aged ≥65 years with acute VTE, followed for a mean of 28 months [13]. TMAO levels were categorized into tertiles. No significant association was observed between TMAO levels and recurrent VTE (adjusted subdistribution hazard ratio for high vs. low tertile: 1.44; 95% confidence interval [CI]: 0.80–2.58; *p* = 0.221) or bleeding events. However, a U-shaped association between TMAO and mortality was identified, with the lowest risk at approximately 4 μmol/L. Compared with the lowest tertile, the adjusted hazard ratio for mortality was 0.68 (95% CI: 0.47–0.98; *p* = 0.039) for the middle tertile and 1.02 (95% CI: 0.68–1.52; *p* = 0.922) for the highest tertile. In a case–control metabolomic analysis, 42 VTE patients and 42 matched controls were included [14]. A total of 512 metabolites grouped into 62 biological pathways were identified. A 21-metabolite panel discriminated VTE patients from controls with an area under the curve (AUC) of 0.92 (selectivity 0.857; sensitivity 0.971). Twenty-five biological functional groups were significantly altered (*p* < 0.05). TMAO remained associated with residual thrombosis at 3 months. Another case–control study analyzed 54 VTE patients and 57 controls [15]. Plasma levels of TMAO, gamma-butyrobetaine (γBB), and trimethyllysine were measured using ultra-high-performance liquid chromatography–mass spectrometry. No significant differences were observed between groups. Weak correlations were identified between these metabolites and thrombin generation parameters. Ząbczyk et al. performed a prospective study of 120 normotensive patients with acute PE. Median LPS levels at admission were 70.5 pg/mL [17]. The patients in the highest LPS quartile (≥82 pg/mL) showed increased thrombin generation (+18.6%), reduced fibrin clot permeability (−14.5%), and prolonged clot lysis time (+25.3%). LPS levels decreased by 40.4% after 3 months of anticoagulation. LPS correlated with zonulin (r = 0.66; *p* < 0.0001), suggesting increased intestinal permeability. No associations were observed between LPS and fibrin parameters. Finally, a case–control study included eight patients with VTE and seven healthy controls [18]. Gut microbiota composition was assessed through 16S ribosomal RNA gene sequencing, and serum metabolomics was assessed through liquid chromatography–mass spectrometry. Significant differences in alpha and beta diversity were observed. VTE patients showed enrichment of *Blautia*, *Roseburia*, *Coprococcus*, and *Ruminococcus*. Alterations in choline and lithocholic acid levels were identified, with a positive correlation between these metabolites and microbial abundance.

#### 3.2.2. Mendelian Randomization Studies

Eight studies used MR to examine causal relationships between gut microbiota and VTE. This first published study investigated the potential relationship between gut microbiota and DVT using a genetic epidemiology approach) [16]. Genome-wide association study (GWAS) summary data for DVT (UK Biobank, N = 9059) and gut microbiota (Flemish Gut Flora Project and German cohorts; total N = 3890) were analyzed. The analysis identified suggestive genetic correlations between DVT and several microbial taxa, including *Streptococcaceae*, *Streptococcus*, *Lactobacillales*, and *Dialister*. MR analysis demonstrated a statistically significant difference between *Streptococcaceae* and DVT risk (OR 1.005; *p* = 0.027). Sensitivity analyses showed no evidence of heterogeneity or horizontal pleiotropy. Xu et al. performed a bidirectional two-sample MR study using MiBioGen data (n = 18,340) and Integrative Epidemiology Unit Open genome-wide association study datasets (up to 361,194 individuals) [19]. Seven suggestive causal relationships were identified for lower extremity DVT and five for DVT combined with PE. The genus *Butyricicoccus* was associated with reduced risk, whereas *Clostridium innocuum* was associated with increased risk. Sensitivity analyses, including MR-Egger and weighted median methods, were performed. Another two-sample MR study evaluated the suggestive causal relationship between gut microbiota and VTE using genetic data from the MiBioGen consortium (exposure) and the UK Biobank (outcome) [20]. Multiple MR methods were applied, including inverse-variance weighted (IVW), weighted median, MR-Egger, simple mode, and weighted mode, with sensitivity analyses performed using Cochran’s Q test, MR-PRESSO, and the MR-Egger intercept. At the genus level, five microbial taxa showed suggestive causal associations with VTE. *Coprococcus* was associated with an increased risk of VTE (odds ratio [OR]: 1.00; 95% CI: 1.00–1.00; *p* = 0.02). In contrast, *Slackia* (OR: 0.99; 95% CI: 0.99–0.99; *p* = 0.02), *Butyricicoccus* (OR: 0.99; 95% CI: 0.99–0.99; *p* = 0.03), *Eubacterium coprostanoligenes* group (OR: 0.99; 95% CI: 0.99–0.99; *p* = 0.04), and Bacteroides (OR: 0.99; 95% CI: 0.99–0.99; *p* = 0.02) were associated with a reduced risk of VTE. A Chinese study performed a two-sample MR analysis using MiBioGen and FinnGen datasets, evaluating arterial thromboembolism and VTE separately [21]. Sixteen microbial genera were associated with thromboembolic outcomes. For VTE, *Ruminococcaceae* groups and *Sutterella* showed protective associations, whereas *Eubacterium rectale* was associated with increased risk; however, none remained statistically significant after correction for multiple testing. Cen et al. investigated the suggestive causal relationship between gut microbiota and PE within the gut–lung axis [22]. Genetic variants associated with gut microbiota were obtained from genome-wide association studies (GWASs), including 18,340 individuals, and used as instrumental variables. The outcome data were derived from the IEU Open GWAS project, including 2118 PE cases and 359,076 controls. At the genus level, four microbial taxa were associated with a reduced risk of PE. Specifically, *Slackia* (*p* = 0.031), *Oscillospira* (*p* = 0.038), *Bacteroides* (*p* = 0.032), and *Clostridium sensu stricto* (*p* = 0.049) showed protective associations. Huang et al. conducted a two-sample MR study to investigate the potential association between gut microbiota and VTE across multiple taxonomic levels (phylum, class, order, family, and genus) [23]. Genetic variants associated with gut microbiota abundance were obtained from large GWAS meta-analysis data and used as instrumental variables after the exclusion of pleiotropic variants using PhenoScanner and MR-PRESSO. The primary analysis was conducted using inverse-variance weighting, complemented by the weighted median, MR-Egger, simple median, and MR-PRESSO methods. Sensitivity analyses, reverse MR, and multivariable MR were performed to assess robustness. The *Firmicutes phylum* was identified as robustly protective against VTE. Additionally, five taxa within the *Actinobacteria phylum* (*Bifidobacteriales* order, *Actinomycetales* order, *Bifidobacteriaceae* family, *Actinomycetaceae* family, and *Slackia* genus) and two taxa within the *Firmicutes phylum* (*Bacillales* order and *Lachnospiraceae UCG-010* genus) were suggestively associated with reduced VTE risk. In contrast, three taxa within the *Firmicutes phylum* (*Bacilli* class, *Lactobacillales* order, and *Lactococcus* genus) were suggestively associated with increased VTE risk. Multivariable MR analysis identified independent associations involving the *Firmicutes phylum* and the *Lachnospiraceae UCG-010* genus. Another two-sample MR study evaluated the causal effects of gut microbiota and plasma metabolites on VTE [24]. Genetic instruments for 211 gut microbiota taxa and 489 plasma metabolites were used to infer causality. The analysis was complemented by mediation and pathway analyses, as well as reverse MR to explore bidirectional relationships. Sixteen gut microbiota taxa were identified as causally associated with thromboembolic outcomes, including protective taxa such as *Firmicutes* and *Clostridia* and risk-associated taxa such as *Bacteroidetes* and *Desulfovibrionaceae*. In addition, 40 plasma metabolites showed suggestive causal relationship with DVT or PE. Reverse MR analysis identified 11 changes in gut microbiota potentially secondary to thrombotic events. Mediation analysis identified 10 metabolite-related pathways, including arginine biosynthesis, linking gut microbiota to thrombotic risk. Finally, Wang et al. performed a mediation MR analysis incorporating 207 microbial taxa, 205 bacterial pathways, and 731 immune cell traits [25]. Sixteen bacterial traits were suggestively associated with VTE. Three taxa were found to influence VTE through two immune cell populations. Specifically, the class *Bacteroidia* was associated with reduced VTE risk, mediated by double-negative (CD4^−^ CD8^−^) natural killer T cells.

### 3.3. Risk of Bias

Risk of bias was assessed according to study design. Observational studies were evaluated using the Newcastle–Ottawa Scale (NOS) (Table 3), while MR studies were assessed using a structured qualitative approach based on instrumental variable assumptions (Table 4).

#### 3.3.1. Observational Studies

Reiner et al. [13] presented the lowest risk of bias, given its prospective multicenter design, objective confirmation of VTE, blinded biomarker assessment, and structured follow-up. Fraser et al. [14] showed a moderate risk of bias, mainly related to selection bias in the control group, which included individuals with a family history of VTE. Canyelles et al. [15] also showed a moderate risk of bias, as, despite appropriate case–control selection and matching, comparability was limited and residual confounding could not be excluded. Similarly, Ząbczyk et al.’s study [17] was considered to be at a moderate risk of bias, as although it was a prospective study with standardized measurements, the absence of an external comparison group limited comparability. Finally, Fan et al. [18] were considered to be at moderate risk due to their cross-sectional design, small sample size, and lack of adjustment for relevant factors, such as diet. Overall, the risk of bias across observational studies ranged from low to moderate, with the main limitations related to confounding, selection bias, and heterogeneity in study design.

#### 3.3.2. Mendelian Randomization Studies

Yang et al. [16]’s and Xu et al.’s studies [19] were considered to be at moderate risk of bias due to incomplete reporting of key parameters, including the absence of F-statistics and unclear reporting of heterogeneity. In contrast, Xi et al. [20], Cen et al. [22], and Huang et al. [23] were considered to have a low risk of bias, as they consistently applied multiple MR methods, formally assessed pleiotropy using MR-Egger and/or MR-PRESSO, and reported no evidence of pleiotropy or significant heterogeneity. In contrast, Wang et al. [21] showed a moderate risk of bias, as reporting of pleiotropy and heterogeneity assessments was limited despite the use of MR-Egger-based methods. The Cheng et al. [24] study was classified as low risk of bias, explicitly reported F-statistics greater than 10, supported adequate instrument strength, and performed comprehensive sensitivity analyses, including MR-Egger and MR-PRESSO, with no evidence of pleiotropy or heterogeneity. Finally, Wang et al.’s study [25] was classified as having a moderate risk of bias, as although it included extensive sensitivity analyses and mediation MR, reporting of instrument strength was incomplete and quantitative details of some sensitivity analyses were not consistently provided. Overall, the risk of bias across MR studies ranged from low to moderate, with the main limitations related to incomplete reporting of instrument strength and heterogeneity.

The overall certainty of the evidence across outcomes was low across all evaluated domains (Table 5).

## 4. Discussion

This systematic review aimed to evaluate the relationship between gut microbiota dysbiosis and VTE. Consequently, it focused on studies investigating alterations in gut microbiota composition with potential prothrombotic effects.

The gut microbiota is emerging as a key player in the pathogenesis of VTE through multiple mechanisms, including the production of bacterial metabolites, the translocation of LPS, and the modulation of intestinal barrier function. Current evidence suggests that both dysbiosis and specific bacterial taxa may influence thrombotic risk through proinflammatory and procoagulant effects.

The role of LPS as a link between gut dysbiosis and VTE is biologically plausible but currently supported by limited clinical evidence. LPS provides a coherent mechanistic framework connecting intestinal dysbiosis with coagulation activation. Experimental data demonstrate that LPS simultaneously activates both the tissue factor and contact pathways [26,27,28]. Notably, intracellular sensing of LPS via caspase-11 leads to gasdermin D-mediated membrane permeabilization, calcium influx, and phosphatidylserine exposure, thereby providing a procoagulant surface for TF–factor VII complex assembly [26]. In parallel, aggregated LPS structures promote factor XII activation, reinforcing thrombin generation through the intrinsic pathway [27,28]. These pathways converge on a procoagulant phenotype and offer strong biological plausibility for endotoxemia’s causal role in VTE. However, despite this robust mechanistic rationale, clinical data remain scarce. Importantly, this mechanistic model is supported by clinical observations. In patients with acute PE, higher circulating LPS levels were associated with increased thrombin generation, reduced fibrin clot permeability, and prolonged clot lysis time, indicating a shift toward a denser, less permeable, and hypofibrinolytic clot structure [28]. The concomitant elevation of plasminogen activator inhibitor-1 (PAI-1) further supports the notion that impaired fibrinolysis is a downstream effect of endotoxemia [17]. Moreover, the observed reduction in LPS levels following anticoagulation suggests that endotoxemia is not merely a static marker but may be dynamically linked to the thrombotic state [17]. Nevertheless, these findings derive from a single observational study [17] and therefore should be interpreted with caution.

TMAO is among the most extensively studied gut microbiota-derived metabolites in cardiovascular disease, with strong experimental evidence supporting a prothrombotic role. Studies have shown that TMAO enhances platelet activation by increasing intracellular calcium release, thereby amplifying agonist-induced platelet responsiveness [29]. In vivo models further demonstrate that both direct TMAO administration and microbiota-dependent TMAO generation via dietary precursors accelerate thrombus formation, supporting a causal link between TMAO and arterial thrombosis [29,30]. Additional experimental evidence reinforces this relationship by modulating hepatic flavin monooxygenase 3 (FMO3), the key enzyme responsible for TMAO generation. Genetic and pharmacological manipulation of FMO3 alters systemic TMAO levels and directly impacts platelet reactivity and thrombotic potential in vivo [31]. However, clinical evidence in VTE remains inconsistent. A large prospective cohort study of patients with acute VTE did not demonstrate a significant association between circulating TMAO levels and recurrent thrombotic events or bleeding complications [13]. Instead, a non-linear, U-shaped relationship between TMAO and mortality was observed, suggesting that both low and high TMAO levels may be associated with adverse outcomes. These findings are supported by additional case–control data showing no significant differences in TMAO or related metabolites, including γ-butyrobetaine and trimethyllysine, between patients with VTE and controls [15]. Moreover, correlations between these metabolites and thrombin generation parameters were weak and, in some cases, directionally inconsistent with a prothrombotic role. Collectively, these results challenge the direct translation of experimental findings into clinical thrombotic risk in VTE populations. Beyond platelet activation, TMAO exerts pleiotropic effects on cholesterol metabolism, bile acid homeostasis, vascular function, and inflammatory pathways, including the activation of the NLRP3 inflammasome [30,32]. These broader systemic effects may contribute to cardiovascular risk in arterial disease, where consistent associations between elevated TMAO levels and adverse outcomes have been reported. However, such associations appear attenuated or absent in VTE, indicating potential differences in pathophysiological relevance between arterial and venous thrombosis.

Bile acids represent an emerging class of gut microbiota-derived metabolites with potentially relevant, yet complex, effects on thrombosis. Secondary bile acids appear to exert dual effects [33,34,35]. On the one hand, metabolites such as lithocholic acid may inhibit platelet activation through TGR5-mediated signaling, involving PKA activation and modulation of AKT/ERK1/2 pathways, suggesting a potential antithrombotic role [36]. On the other hand, certain bile acids can enhance the procoagulant activity of the TF:FVIIa complex, indicating context-dependent prothrombotic effects [33]. Metabolomic studies in patients with VTE have identified alterations in bile acid profiles, including increased lithocholic acid levels, along with the upregulation of bile secretion pathways, suggesting a possible contribution to venous thrombotic disease [18]. Overall, secondary bile acids introduce an additional layer of complexity within the gut–venous thrombosis axis, with effects that are less consistent and more context-dependent than those observed for LPS and TMAO.

In addition, the interaction between gut microbiota, bile acid metabolism, and immune regulation may represent a key integrative axis in the pathogenesis of VTE. Bile acids not only act as metabolic intermediates but also function as signaling molecules through receptors such as FXR and TGR5, thereby modulating inflammatory and coagulation pathways. These signaling networks may influence endothelial function, platelet activation, and thrombin generation. Furthermore, microbiota-driven immune modulation, including alterations in immune-mediated pathways and specific immune cell populations, may contribute to thromboinflammatory responses. This integrated microbiota–metabolite–immune axis provides a conceptual framework that may help explain the heterogeneity observed across studies and highlights potential targets for future mechanistic and interventional research.

Recent studies have demonstrated that patients with VTE exhibit significant alterations in gut microbiota composition, including differences in alpha and beta diversity and enrichment of genera such as *Blautia*, *Roseburia*, *Coprococcus*, and *Ruminococcus* [18]. Additionally, genetic evidence suggests that specific taxa such as *Streptococcaceae* may have a suggestive causal relationship with increased VTE risk [16]. However, these findings are derived from small observational studies and remain heterogeneous, limiting their reproducibility and clinical applicability. MR analyses provide additional insight, identifying taxa with potential protective associations, such as *Ruminococcaceae NK4A214*, *Sutterella*, and *Christensenellaceae*, as well as taxa associated with increased risk, including *Eubacterium rectale* and *Erysipelatoclostridium* [19,21]. Nevertheless, most associations are modest and often lose statistical significance after correction for multiple testing, underscoring the need for cautious interpretation. Beyond compositional changes, emerging evidence suggests that immune-mediated mechanisms may link gut microbiota to thrombosis. Certain taxa, such as *Bacteroidia*, appear to reduce VTE risk by modulating specific immune cell populations, including double-negative natural killer T (NKT) cells [25]. This immunometabolic interaction provides a plausible pathway connecting dysbiosis to systemic inflammation and thrombosis, although its clinical relevance remains to be fully established.

The growing recognition of the gut–VTE axis has opened new avenues for therapeutic intervention, although most of these strategies remain at a preclinical or hypothesis-generating stage. Among the proposed interventions, modulation of the gut microbiota through probiotics and prebiotics is one of the most conceptually appealing strategies. These approaches aim to restore eubiosis and promote the expansion of short-chain fatty acid-producing bacteria, particularly those that generate butyrate and propionate, which have been shown to have anti-inflammatory and immunomodulatory effects [37,38,39]. However, current evidence remains indirect, and no clinical trials have demonstrated that probiotic or prebiotic supplementation reduces the incidence or recurrence of VTE. Postbiotics may represent a particularly promising next-generation approach. Unlike probiotics, postbiotics consist of inactivated microorganisms or their functional components. They may exert effects both locally in the intestine and systemically [40]. Nevertheless, their role in VTE is still largely speculative. Restoration of intestinal barrier integrity is another therapeutic target. Since increased permeability may facilitate translocation of LPS into the bloodstream, strategies aimed at normalizing gut barrier function could theoretically reduce endotoxemia and its downstream procoagulant effects [9]. However, barrier restoration remains difficult to operationalize clinically. Pharmacological inhibition of microbial metabolic pathways is one of the most innovative approaches. In particular, inhibition of microbial trimethylamine (TMA) lyases has emerged as a strategy to reduce TMAO without broadly eradicating commensal bacteria [11,41]. This approach is appealing because it targets a specific prothrombotic metabolite pathway while, in theory, preserving beneficial microbial functions. However, enthusiasm should be tempered by the fact that the clinical relevance of TMAO in venous thrombosis remains inconsistent [11]. Dietary modification is perhaps the most immediately applicable microbiome-targeted intervention. Diets enriched in fiber and polyphenols may promote the generation of beneficial metabolites and favor a less thrombogenic microbiome [37,38]. Reducing dietary precursors of TMAO, such as choline and carnitine, has been proposed as a preventive strategy [9]. The main strength of this approach lies in its feasibility and broad cardiometabolic benefits. However, its limitations are equally important. Dietary effects on the microbiome are individualized and difficult to sustain over time. In addition, indiscriminately reducing choline or carnitine could have unintended metabolic consequences, so any dietary strategy would need to be carefully balanced [9,11]. Fecal microbiota transplantation is a radical approach to modifying the intestinal ecosystem. In theory, these strategies could re-establish microbial communities associated with lower inflammatory and thrombotic risk. Yet, at present, this remains highly speculative in the VTE field. Their clinical use is still largely confined to other conditions, particularly recurrent *Clostridioides difficile* infection, and extrapolation to thrombosis is premature [37]. Traditional Chinese medicine has also been proposed as a microbiome-modulating antithrombotic strategy, with recent studies suggesting beneficial effects on vascular thromboinflammation through regulation of gut microbiota and derived metabolites [42]. Although it is an interesting field, interpretation requires caution. Many studies are heterogeneous in design, involve complex multicomponent interventions, and may be difficult to generalize outside their original clinical setting. Therefore, these formulations should currently be considered exploratory rather than established therapeutic options. Taken together, microbiome-targeted therapies offer an attractive framework for VTE prevention and treatment, especially in settings characterized by chronic inflammation or intestinal barrier dysfunction, such as inflammatory bowel disease [9]. However, the current evidence does not support their incorporation into routine VTE management. Future research should prioritize well-designed randomized trials, ideally integrating microbiome profiling, metabolomics, markers of intestinal permeability, and thrombotic outcomes, to define which patients may benefit, which interventions are biologically meaningful, and whether modulation of the gut microbiota can translate into clinically relevant reductions in VTE risk.

This systematic review has several strengths. First, it provides a more comprehensive and up-to-date synthesis of the available evidence on the relationship between gut microbiota and VTE, integrating findings from observational studies, metabolomic analyses, and MR approaches. Second, the inclusion of MR studies represents a major strength, as it allows exploration of potential causal relationships beyond traditional observational associations, thereby strengthening the biological plausibility of the findings. Third, this review systematically evaluates multiple mechanistic pathways linking gut microbiota to thrombosis, including microbial composition, metabolite production, intestinal permeability, immune modulation, and coagulation activation, offering a multidimensional perspective on VTE pathophysiology. Fourth, a structured and rigorous methodological approach was applied, including predefined inclusion criteria, independent study selection by multiple reviewers, and formal risk-of-bias assessment using validated tools, enhancing the transparency and reproducibility of the review. Finally, this study highlights emerging potentially modifiable pathways, such as microbiota-derived metabolites and immune-mediated mechanisms, which may inform future research and the development of novel therapeutic strategies.

There are some limitations that need to be acknowledged. First, observational studies are characterized by small sample sizes and predominantly case–control or cross-sectional designs, which preclude establishing temporal precedence and increase the risk of selection bias. In particular, differences in the source of case and control groups, variability in matching strategies, and heterogeneity in inclusion criteria across studies may have influenced the observed associations. In some case–control studies, controls may not have been fully representative, potentially introducing bias. In addition, substantial clinical heterogeneity exists across studies, including differences in VTE phenotypes (DVT vs. PE), disease stage (acute vs. stable), and patient populations, which may limit the generalizability of findings. Second, microbiome assessment methods vary considerably, ranging from 16S rRNA sequencing to metabolomic profiling and genetic proxies in Mendelian randomization studies. These methodological differences, together with variability in sample type, sequencing platforms, and analytical pipelines, complicate direct comparisons across studies and may contribute to inconsistent results. Third, residual confounding remains a major concern, particularly in observational studies, where key determinants of gut microbiota composition—such as diet, antibiotic exposure, comorbidities, and lifestyle factors—are not consistently controlled for. Fourth, although MR studies aim to provide supportive evidence for potential causal relationships, they are subject to inherent limitations, including potential horizontal pleiotropy, incomplete reporting of instrument strength, and the use of genetic instruments that may not fully capture the complexity of microbial ecosystems. Moreover, many reported associations did not remain significant after correction for multiple testing. Fifth, most studies lack longitudinal microbiome data before thrombotic events, limiting the ability to disentangle whether dysbiosis is a causal factor, a consequence of VTE, or both. Sixth, discrepancies in findings regarding key metabolites, such as TMAO, may reflect differences in timing of measurements, anticoagulation status, and dietary factors, underscoring the need for standardized protocols. In addition, the integration of risk of bias assessments highlights that the current body of evidence is limited by methodological constraints. Observational studies were mainly affected by potential confounding, selection bias, and heterogeneity in study design, while MR studies frequently showed incomplete reporting of instrumental variables and limited assessment of instrument strength. These factors should be considered when interpreting the findings. Finally, the possibility of reverse causality should be considered. It remains unclear whether gut microbiota dysbiosis is a causal factor in VTE or a consequence of the thrombotic event itself. Acute VTE and its treatment may influence gut microbiota composition through systemic inflammation, hospitalization, changes in diet, or exposure to medications such as anticoagulants. In addition, physiological stress and reduced mobility associated with acute illness may further contribute to microbiota alterations. Therefore, the bidirectional relationship between VTE and gut microbiota cannot be excluded.

The overall certainty of the evidence was assessed qualitatively, taking into account study design, consistency of results, directness of evidence, precision, and risk of bias across studies. Overall, the evidence was considered low to very low in certainty. Observational studies were limited by small sample sizes, heterogeneity in design, and potential residual confounding, leading to low certainty. MR studies provided additional support for potential association; however, most findings were modest in magnitude and not robust after correction for multiple testing; they were therefore also considered of low certainty. The overall body of evidence was further downgraded due to inconsistent study designs and indirectness arising from heterogeneous exposures and outcomes.

## 5. Conclusions

Gut microbiota dysbiosis emerges as a potential contributor to VTE through interconnected mechanisms involving inflammation, intestinal permeability, and microbiota-derived metabolites such as TMAO and LPS. While current evidence supports a biologically plausible link, its clinical relevance remains to be fully established. The identification of microbiome-related pathways and taxa associated with thrombotic risk highlights promising targets for future therapeutic and preventive strategies. However, robust prospective studies with standardized methodologies are required to clarify causality and enable translation into clinical practice.

## Figures and Tables

**Table 1:** Gut microbiota dysbiosis and mechanisms involved in venous thromboembolism.

Component	Healthy State (Eubiosis)	Dysbiotic State	Thrombotic Mechanism
Microbiota composition	↑ Commensal anaerobes (*Butyricicoccus*, Ruminococcaceae, Lachnospiraceae)	↓ Commensal anaerobes; ↑ pathogenic bacteria (Enterobacteriaceae, *Clostridium innocuum*, *Eubacterium rectale*)	Loss of protective bacterial metabolites; overgrowth of prothrombotic species
Intestinal barrier	Intact tight junctions; low permeability; effective barrier function	Disrupted tight junctions; ↑ intestinal permeability (“leaky gut”)	Translocation of bacterial products (LPS) into systemic circulation
LPS/endotoxin	Minimal systemic LPS; contained within gut lumen	↑ Systemic LPS from Gram-negative bacteria (Enterobacteriaceae); gut-derived endotoxemia	LPS binds TLRs → endothelial and platelet activation → coagulation cascade activation
TMAO pathway	Low TMAO production; balanced TMA lyase activity	↑ Dietary choline/carnitine metabolism; ↑ TMA production; ↑ hepatic FMO3 conversion to TMAO	TMAO enhances platelet Ca^2+^ release → platelet hyperreactivity → ↑ thrombosis risk
Short-chain fatty acids (SCFAs)	↑ SCFA production (butyrate, acetate, propionate); anti-inflammatory effects	↓ SCFA production; loss of anti-inflammatory protection	Reduced immune modulation and vascular protection; ↑ systemic inflammation
Bile acid metabolism	Normal bile acid metabolism; balanced primary/secondary bile acids	Altered bile acid metabolism; ↑ lithocholic acid	Dysregulated lipid metabolism and inflammatory signaling
Immune modulation	Balanced immune cell populations; DN NKT cells maintain homeostasis	Altered immune cell populations; dysregulated DN (CD4^−^ CD8^−^) NKT cells	Loss of immune-mediated thrombosis protection
Metabolomic profile	Balanced choline levels; normal metabolite diversity	↑ Serum choline; ↑ lithocholic acid; altered metabolome	Metabolite-mediated prothrombotic signaling pathways
Clinical outcome	Low VTE risk; normal hemostasis	↑ VTE risk; hypercoagulable state; ↑ DVT and PE incidence	Multifactorial prothrombotic state from combined mechanisms

Symbols: ↑ indicates increased levels, abundance, activation, production, permeability, or risk; ↓ indicates decreased levels, abundance, production, activity, or protection. Abbreviations: Ca^2+^, calcium ion; DN NKT cells, double-negative natural killer T cells (CD4^−^ CD8^−^); DVT, deep vein thrombosis; FMO3, flavin-containing monooxygenase 3; LPS, lipopolysaccharide; PE, pulmonary embolism; SCFAs, short-chain fatty acids; TLR, Toll-like receptor; TMA, trimethylamine; TMAO, trimethylamine N-oxide; VTE, venous thromboembolism.

**Table 2:** Main characteristics, exposures, methodological features and outcomes of the included studies.

Author (Year)	Scope/Objectives	Country	N	Patient Groups	Design	Exposure Type	Outcome Definition	Methodology	Adjustment for Confounders	Main Results
Reiner et al. (2019) [13]	TMAO and recurrent VTE/mortality	Switzerland	859	≥65 years acute VTE	Prospective cohort	Metabolites (TMAO)	Recurrent VTE, bleeding, mortality (objectively confirmed)	HPLC TMAO; follow-up 28 months	Yes	U-shaped mortality; no association with recurrence/bleeding
Fraser et al. (2020) [14]	Metabolic profile in VTE	New Zealand	84	42 VTE, 42 controls	Case–control	Metabolites	VTE vs. controls (post-event)	LC-MS metabolomics	Yes	21-metabolite panel (AUC 0.92); TMAO associated
Canyelles et al. (2022) [15]	TMAO, γBB, TML in VTE	Spain	111	54 VTE, 57 controls	Case–control	Metabolites	VTE vs. controls (NR for definition)	UHPLC-MS; coagulation assays	NR	No differences; correlations with thrombin generation
Yang et al. (2022) [16]	Genetic correlation and suggestive causal relationship between gut microbiota and DVT	European cohorts: United Kingdom, Belgium, Germany	DVT GWAS: 452,264; Microbiota GWAS: 3890	DVT GWAS: 452,264 (9059 cases, 443,205 controls); Microbiota GWAS: 3890	Two-sample MR study using GWAS summary data	Genetically predicted gut microbiota taxa	DVT	LDSC + Mendelian Randomization (IVW, MR-Egger, weighted median)	Yes (SNP selection, exclusion of confounder-associated variants, MR assumptions)	Suggestive genetic correlations; causal association for *Streptococcaceae*
Ząbczyk et al. (2023) [17]	LPS in PE	Poland	120	120 PE patients	Prospective cohort	Microbial products (LPS)	Acute PE (clinically confirmed)	Serial biomarkers + coagulation	NR	Higher LPS → prothrombotic profile
Fan et al. (2024) [18]	Microbiota + metabolome in VTE	China	15	8 VTE, 7 controls	Case–control	Microbiota + metabolites	VTE vs. controls (NR)	16S rRNA + LC-MS	No	Dysbiosis + altered bile acid metabolism
Xu et al. (2024) [19]	GM and LEDVT/PE	China	18,340/361,194	GWAS	MR	Genetic liability to microbiota	LEDVT, LEDVT + PE (GWAS-based)	IVW + sensitivity MR	NR	Butyricicoccus protective; Clostridium risk
Xi et al. (2024) [20]	GM and VTE	China	18,340/UK Biobank	GWAS	MR	Genetic liability to microbiota	VTE (GWAS-based)	IVW, MR-Egger, PRESSO	NR	5 taxa associated; no pleiotropy
Wang (2025) [21]	GM and thromboembolism	China	18,340/FinnGen	GWAS	MR	Genetic liability to microbiota	AET, VTE, PE (GWAS-based)	IVW + sensitivity MR	NR	16 genera; not significant after correction
Cen et al. (2025) [22]	GM and PE	China	18,340/361,194	GWAS	MR	Genetic liability to microbiota	PE (GWAS-based)	IVW + sensitivity MR	NR	4 genera associated; no pleiotropy
Huang et al. (2025) [23]	GM and VTE	China	18,340/large GWAS	GWAS	MR	Genetic liability to microbiota	VTE (GWAS-based)	IVW, MR-Egger, MVMR	NR	Firmicutes protective
Cheng et al. (2025) [24]	GM + metabolites and VTE	China	18,340/7824	GWAS	MR	Microbiota + metabolites	VTE, DVT, PE (GWAS-based)	IVW, MR-Egger, PRESSO, mediation	NR	16 taxa + 40 metabolites; mediation pathways
Wang et al. (2026) [25]	GM–immune–VTE	China	18,340/731 traits	GWAS	MR	Microbiota + immune traits	VTE (GWAS-based)	IVW, MR-Egger, Bayesian MR	NR	Mediation via immune cells

Abbreviations: AET, arterial thromboembolism; AUC, area under the curve; Ca^2+^, calcium ion; CLT, clot lysis time; DN NKT cells, double-negative natural killer T cells; DVT, deep vein thrombosis; ETP, endogenous thrombin potential; GM, gut microbiota; GWAS, genome-wide association study; HPLC, high-performance liquid chromatography; IQR, interquartile range; IVW, inverse variance weighted; LC-MS, liquid chromatography–mass spectrometry; LEDVT, lower extremity deep vein thrombosis; LDSC: Linkage Disequilibrium Score Regression; LPS, lipopolysaccharide; MR, Mendelian randomization; MR-Egger, Mendelian randomization Egger regression; MR-PRESSO, Mendelian Randomization Pleiotropy RESidual Sum and Outlier; MVMR, multivariable Mendelian randomization; n-3 PUFAs, omega-3 polyunsaturated fatty acids; NR, not reported; PE, pulmonary embolism; SNP, single-nucleotide polymorphism; TMAO, trimethylamine N-oxide; TML, trimethyllysine; UHPLC-MS, ultra-high-performance liquid chromatography–mass spectrometry; VTE, venous thromboembolism; γBB, gamma-butyrobetaine.

**Table 3:** Risk of bias assessment of observational studies.

Study	Selection (Max 4)	Comparability (Max 2)	Outcome/Exposure (Max 3)	Total (Max 9)	Risk of Bias	Key Justification
Reiner et al. (2019) [13]	3	1	3	7	Low	Large multicenter cohort; objective outcomes; blinded exposure assessment; internal comparison
Fraser et al. (2020) [14]	2	1	2	5	Moderate	Controls with family history of VTE; good metabolomics assessment; possible selection bias
Canyelles et al. (2022) [15]	3	1	2	6	Moderate	Cases and controls selected from RETROVE cohort; matched by age/sex; objective VTE diagnosis; limited adjustment for confounders
Ząbczyk et al. (2023) [17]	3	0	3	6	Moderate	No external comparison group; detailed lab assessment; repeated measures
Fan et al. (2024) [18]	3	1	2	6	Moderate	Small sample; matched controls; robust microbiome/metabolomics methods; cross-sectional design

Abbreviations: VTE, venous thromboembolism.

**Table 4:** Risk of bias assessment of Mendelian randomization studies.

Study	Instrument Strength (F-Statistic)	Confounding/Population Stratification	Horizontal Pleiotropy (Egger/PRESSO)	Heterogeneity (Cochran’s Q)	Sensitivity Analyses Consistency	Risk of Bias
Yang et al. (2022) [16]	NR	Addressed (SNP selection, exclusion of confounders via PhenoScanner; European ancestry reduces stratification)	Not detected (MR-Egger *p* > 0.05; MR-PRESSO global test *p* > 0.05)	Not detected (Cochran’s Q *p* > 0.05)	Generally consistent for Streptococcaceae (IVW and weighted median significant; others non-significant)	Moderate
Xu et al. (2024) [19]	NR	Likely low (GWAS-based, MiBioGen)	Assessed (Egger, sensitivity)	Assessed	Yes (IVW + others)	Moderate
Xi et al. (2024) [20]	NR	Likely low	Assessed (Egger, MR-PRESSO)—no pleiotropy	Assessed	Yes	Low
Wang et al. (2025) [21]	NR	Likely low	Assessed (Egger, sensitivity)	NR	Yes	Moderate
Cen et al. (2025) [22]	NR	Likely low	Assessed—no pleiotropy	Assessed—no heterogeneity	Yes	Low
Huang et al. (2025) [23]	NR	Likely low (multi-cohort GWAS)	Assessed (Egger, PRESSO)—no pleiotropy	Assessed—no heterogeneity	Yes	Low
Cheng et al. (2025) [24]	>10	Likely low	Assessed (Egger, PRESSO)—no pleiotropy	Assessed	Yes (multiple MR methods)	Low
Wang et al. (2026) [25]	NR	Likely low	Assessed (Egger, PRESSO)	Assessed (Cochran Q)	Yes (including mediation MR)	Moderate

Abbreviations: GWAS, genome-wide association study; IVW, inverse variance weighted; MR, Mendelian randomization; MR-Egger, Mendelian randomization Egger regression; MR-PRESSO, Mendelian Randomization Pleiotropy RESidual Sum and Outlier; NR, not reported; SNP, single-nucleotide polymorphism.

**Table 5:** Strength of the evidence across outcomes.

Outcome	Study Design	Risk of Bias	Inconsistency	Indirectness	Imprecision	Certainty
Gut microbiota composition and VTE	Observational + Mendelian randomization studies	Moderate	High	Moderate	High	Low
Microbial metabolites (TMAO, LPS) and VTE	Observational studies	Moderate	High	Moderate	High	Low
Specific microbial taxa	Mendelian randomization studies	Low–moderate	High	Moderate	High	Low

Abbreviations: LPS, lipopolysaccharide; TMAO, trimethylamine N-oxide; VTE, venous thromboembolism.

## Data Availability

The original contributions presented in this study are included in the article. Further inquiries can be directed to the corresponding author.

## Abbreviations

The following abbreviations are used in this manuscript:

DVT	deep vein thrombosis
GWAS	genome-wide association study
LPS	lipopolysaccharide
MR	Mendelian randomization
PE	pulmonary embolism
TMAO	trimethylamine -N-oxide
VTE	venous thromboembolism
